# Napping and cognitive decline: a systematic review and meta-analysis of observational studies

**DOI:** 10.1186/s12877-022-03436-2

**Published:** 2022-09-15

**Authors:** Celia Álvarez-Bueno, Arthur Eumann Mesas, Sara Reina-Gutierrez, Alicia Saz-Lara, Estela Jimenez-Lopez, Vicente Martinez-Vizcaino

**Affiliations:** 1 Universidad de Castilla-La Mancha, Health and Social Care Research Center, 16071 Cuenca, Spain; 2 Universidad Politécnica Y Artística del Paraguay, Asunción, 001518 Paraguay; 3 Postgraduate Program in Public Health, Universidade Estadual de Londrina, Londrina, Parana, Brasil; 4 Department of Psychiatry Hospital, Virgen de La Luz, Cuenca, Spain; 5 CIBERSAM (Biomedical Research, Networking Centre in Mental Health), Barcelona, Spain; 6 Faculty of Health Sciences, Universidad Autónoma de Chile, 1670 Talca, Chile

**Keywords:** Sleep, Napping, Cognition, Elderly, Review, Meta-analysis

## Abstract

**Background:**

No clear evidence is available for the influence of napping on cognitive function in older adults. This systematic review and meta-analysis aimed to elucidate the cross-sectional and longitudinal relationships between napping and cognitive function (global cognition and memory) and to explore whether some individual characteristics and sleep characteristics can modify this relationship.

**Methods:**

We systematically searched Medline (via PubMed), Web of Science, and Scopus. DerSimonian and Lair and Hartung-Knapp-Sidik-Jonkman random effects methods were used to compute pooled estimates of odds ratios (ORs) and their respective 95% confidence intervals (95% CIs) for the association of global cognition and memory with napping. The mean age, the night sleep time (hours), and the percentage of women, no nappers, and people in the less night-time sleep duration category were used for meta-regressions.

**Results:**

Twenty-five studies were included in this systematic review and meta-analysis, 18 cross-sectional and seven longitudinal studies, including 95,719 participants older than 60 years. The pooled ORs from the cross-sectional analyses were 1.03 (95% CI: 1.01 to 1.06) for global cognition and 1.06 (95%: 0.90 to 1.26) for memory. The pooled ORs from the longitudinal analyses were 1.00 (95% 0.85 to 1.18) for global cognition and 1.08 (95% 0.98 to 1.19) for memory. These associations were not modified by individual or sleep characteristics.

**Conclusion:**

Our data confirm the absence of association between napping and global cognition and memory regardless of the characteristics of the population. This information might be considered when providing lifestyle recommendations to adults with and without cognitive complaints.

**Supplementary Information:**

The online version contains supplementary material available at 10.1186/s12877-022-03436-2.

## Introduction

Aging is accompanied by several physical, social, and psychological changes [1]. These changes may lead to chronic diseases such as dementia and Alzheimer’s disease, the estimated costs and prevalence of which have been increasing in recent years [2]. Due to the limited effects demonstrated by pharmacological treatments, new approaches are needed to address the increasing incidence of cognitive decline and dementia diseases [3]. Thus, nonpharmacological preventive and therapeutic strategies have become key tools to manage cognitive decline and dementia due to their feasibility and safety [4, 5].

Among nonpharmacological treatments, the management of health parameters described as risk factors for the development of dementia has been considered an important element [6]. Additionally, some behaviors, including but not limited to sleep patterns, have been reported to potentially be related to cognitive function [7]. Although changes in the structure and organization of sleep time increase with aging and with the incidence of chronic diseases [8, 9], the direction of the relationship between sleep disorders and cognitive decline is still not clear.

A nap is defined as a short sleep episode typically during daylight hours, the duration of which can range from a few minutes to several hours, while the frequency can vary from occasional naps to several naps daily [10]. Although nap characteristics such as the duration, frequency, intention, and depth of napping might be considered, [11, 12] positive effects of napping have been reported for physical health, cognitive function, and mood [8]. Thus, napping for enjoyment or replacement reasons has been established as an approach to maintain physical activity levels and improve performance [13, 14]. Moreover, excessive daytime napping might be associated with sedentary behaviors and depletion of leisure time activities [15], which may in turn negatively impact cognitive function [8], although not all cognitive domains are equally affected by napping [15]. Additionally, some individual characteristics, including sex [16] and age [17], may influence the relationship between napping and cognitive function.

As a previous narrative review claims, more research is urgently needed to investigate the influence of napping on health in older adults [18]. Therefore, this systematic review and meta-analysis was aimed to quantify the cross-sectional and longitudinal relationships between napping and cognitive function among the general population, distinguishing between global cognition and memory, and to explore whether some individual and sleep characteristics can modify this relationship.

## Methods

We conducted this systematic review and meta-analysis following the PRISMA 2020 statement: an updated guideline for reporting systematic reviews (Supplementary Table 1) [19] and the Cochrane Collaboration Handbook [20]. The protocol for this systematic review and meta-analysis has been previously registered on PROSPERO: CRD42021232071.

### Data sources and searches

A literature search was performed in Medline (via PubMed), Web of Science, and Scopus to identify studies on the association between napping and cognitive function among the adult general population through August 6, 2022. The search strategy combined the following terms: “napping”, “siesta”, “nap”, “nap sleep”, “nap time”, “day sleep”, “daytime sleep”, “daytime nap”, “daytime napping”, “day time sleep”, “day time nap”, “day time napping”, “day-time sleep”, “day-time nap”, “day-time napping”, “elderly”, “older adults”, “older adult”, “middle-aged adults”, “aged individuals”, “aged adults”, senior*, “ancient”, “aging”, “cognition,” “executive,” “executive function,” “cognitive control,” “memory,” “attention,” “metacognition,” “life skills,” “goal setting,” “problem solving,” “self-regulation,” “brain development,” “brain health,” and “neural” (Supplementary Tables 2). We completed the literature search by reviewing the reference lists of the included studies for any further relevant study.

### Study selection

This systematic review includes cross-sectional and longitudinal studies on the relationship between napping and cognitive function among adults. The inclusion criteria were as follows: (1) participants: general population which mean age was older than 60; (2) exposure: napping; and (3) outcome: cognitive function measured using standardized tests.

Studies were excluded when they were (1) focused on children or adolescents, (2) focused on specific populations such as people with dementia or Parkinson’s disease, (3) focused on how progressive cognitive decline could influence daily sleep duration and frequency, or (4) written in languages other than English, French, Portuguese, or Spanish.

The cognitive functions most consistently reported were i) global cognition using the Mini-Mental State Examination (MMSE), Montreal cognitive assessment (MoCA), Trail Making Test (TMT) -A or B, figure drawing, and clock drawing tests; and ii) memory using the Logical Memory II (LM-II), Controlled Oral Word Associated Test (COWAT), and word recall tests. Other cognitive functions measured included inhibition, executive functions, psychomotor speed, self-reported cognitive difficulties, and visuospatial reasoning.

### Data extraction and quality assessment

The main characteristics of the included studies are summarized in tables, including information on (1) subject characteristics (i.e., sample size, the percentage of women, the mean age, and depressive symptoms), (2) exposure (i.e., the device used to measure napping, the total night-time sleep duration, and the total napping time or frequency as reported by original studies), and (3) outcome information (i.e., tests used to measure cognitive function and cognitive domains). Covariates included in the analyses reported by the included studies were summarized in an additional table.

The Quality Assessment Tool for Observational Cohort and Cross-Sectional Studies [21] was used to evaluate the risk of bias. This tool evaluates 14 criteria for longitudinal studies; for cross-sectional designs only 11 were applied. Each criterion can be scored as “yes” when the study achieves the criterion or “no” when the study does not achieve the criterion. Criteria could also be scored as “not reported” when studies did not clearly report the required information.

The literature search, data extraction, and risk of bias assessment were independently performed by 2 researchers (C.A.-B. and A.E.-M.), and disagreements were resolved by consensus or involving a third researcher (V.M.-V.).

### Data synthesis and statistical analysis

To perform the meta-analysis, measures of the association between napping and cognitive function were included in the analysis. We considered only two domains for the statistical analysis, namely, global cognition and memory, for which cross-sectional and longitudinal association analyses were separately conducted. These two domains were the ones most consistently reported across studies, and both were managed for this meta-analysis as reported by the original studies. Meta-analyses or graphical representation for other domains could not be conducted as not enough data were available.

Both the DerSimonian [22] and Lair and Hartung-Knapp-Sidik-Jonkman [23] random effects methods were used to compute pooled estimates of odds ratios (ORs) and their respective 95% confidence intervals (95% CIs) for the association of global cognition and memory with napping. Inconsistency across studies [24, 25] was assessed using the I2 statistic, whose values were considered as follows: not important (0–40%), moderate (30–60%), substantial (50–90%), and considerable (75–100%). Moreover, the corresponding *P* values were also considered [22]. In addition, heterogeneity [26] was evaluated by the τ^2^ statistic, which was interpreted as low when τ^2^ was lower than 0.04, moderate when τ^2^ ranged from 0.04 to 0.14, and as substantial when τ^2^ ranged from 0.14 to 0.40 [27].

When studies provided ≥ 2 measurements for the same cognitive domain using different tests (e.g., immediate and delayed word recall for memory), these measurements were combined to calculate a single pooled OR for the corresponding domain. For the analyses, we considered the data adjusted by the largest number of covariates. When regression models were presented, only those using “no nappers” as reference were considered for the analyses. When studies reported associations by group, data were included as different cohorts in the analyses. Finally, when studies provided a linear regression b coefficient, it was used to calculate OR values [28].

Sensitivity analyses were performed excluding studies one by one from the pooled estimates to evaluate whether any particular study modified the original summary estimate. Meta-regressions were calculated on the basis of sample characteristics: the mean age, the night sleep time (hours), and the percentage of women, no nappers, and people in the less night-time sleep duration category. Finally, small study effects were estimated using Egger’s test [29].

## Results

### Systematic review

The literature search retrieved 982 studies, 25 of which were included in this systematic review and meta-analysis [30–54] (Supplementary Fig. 1). The cross-sectional analysis included 18 studies published from 1996 to 2022, reporting data from 82,757 participants older than 60 years. Furthermore, the seven longitudinal studies reported data for 12,962 participants older than 64 years. Each longitudinal study reported a different follow-up period ranging from 6 months to 11 years. The population reported a total sleep duration ranging from less than 5 h to more than 9 h. The characteristics of napping were diversely reported by the studies, including i) the napping time, ii) the percentage of nappers, iii) the frequency of napping in days per week, and iv) the intentionality (intentional or unintentional) of napping (Supplementary Tables 3 and 4).

Finally, a different set of covariates was used to adjust the analyses reported by the included studies (Supplementary Table 5).

### Risk of bias

After assessing the risk of bias by the Quality Assessment Tool for Observational Cohort and Cross-Sectional Studies, cross-sectional studies met 7 to 8 criteria, and longitudinal studies met 9 to 11 criteria. No study reported information on the sample size calculation or for the blinded assessment of participants. Moreover, four cross-sectional studies reported a participation rate of eligible persons lower than 50%, and three longitudinal studies presented a loss of follow-up after baseline higher than 20% (Supplementary Table 6).

### Meta-analysis

**Fig. 1:**
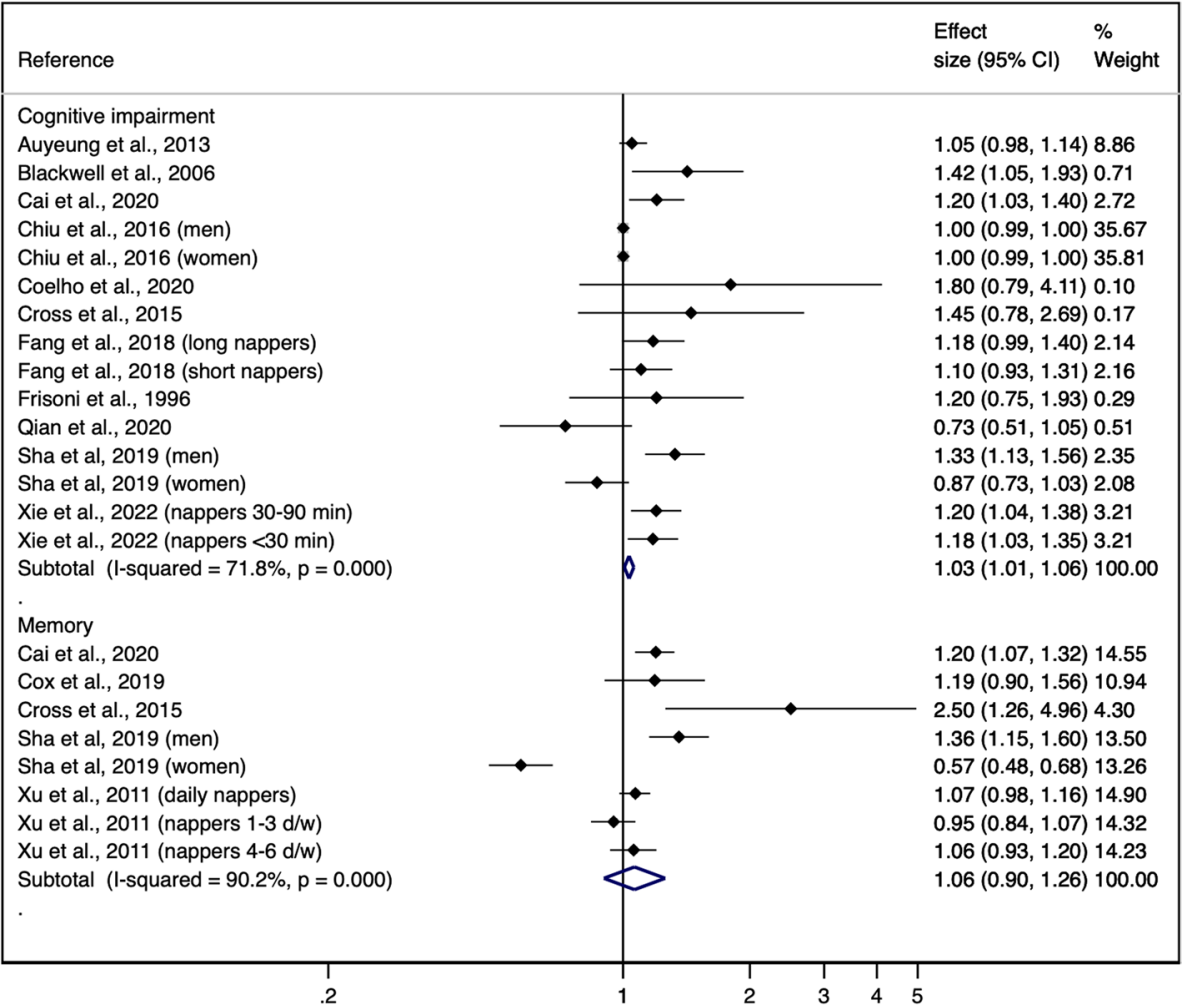
Forest plot for the cross-sectional association between napping and cognitive function domains. ES indicates effect size

**Fig. 2:**
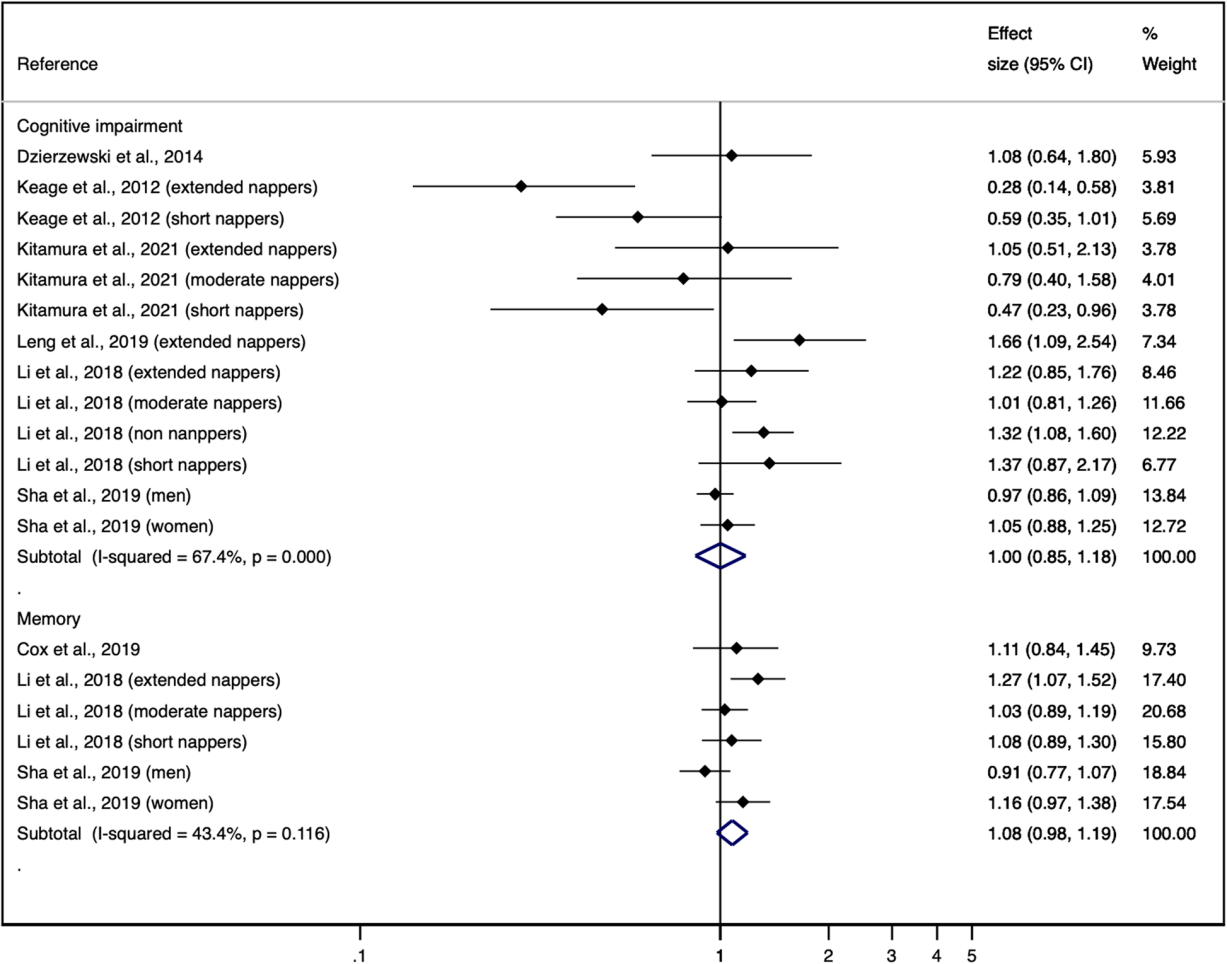
Forest plot for the longitudinal association between napping and cognitive function domains. ES indicates effect size

Using the DerSimoniand and Lair random effect models, the pooled ORs from the cross-sectional analyses were 1.03 (95% CI: 1.01 to 1.06) for global cognition and 1.06 (95%: 0.90 to 1.26) for memory. The pooled ORs from the longitudinal analyses were 1.00 (95% 0.85 to 1.18) for global cognition and 1.08 (95% 0.98 to 1.19) for memory (Figs. 1 and 2). Similar results were obtained using the Hartung-Knapp-Sidik-Jonkman random effect models, the pooled ORs from the cross-sectional analyses were 1.10 (95% CI: 0.99 to 1.20) for global cognition and 1.08 (95%: 0.81 to 1.34) for memory. The pooled ORs from the longitudinal analyses were 0.94 (95% 0.73 to 1.15) for global cognition and 1.07 (95% 0.96 to 1.18) for memory (Supplementary Table 7).

Data for inconsistency and heterogeneity for both models are presented in Supplementary Table 7.

### Meta-regression and sensitivity analysis

Meta-regression analyses indicated that none of the considered characteristics (i.e., the mean age, the night sleep time (hours), and the percentage of women, no nappers, and people in the less nighttime sleep duration category) influence the cross-sectional or longitudinal relationships between napping with global cognition and memory (Supplementary Tables 8 and 9).

Sensitivity analysis revealed that the pooled ORs were modified after removing from the: i) cross-sectional analysis of napping and global cognition, the men and women cohorts included in Chiu et al. study; and ii) cross-sectional analysis of napping and memory and the longitudinal analysis of napping and memory, the women cohort of Sha et al. study (Supplementary Tables 10 and 11).

### Small study effects

Small study effects was observed for the cross-sectional analysis of the association between napping and global cognition (Supplementary Table 12).

## Discussion

This study aimed to assess the cross-sectional and longitudinal associations between napping and cognitive function among older adults. Our data suggest no association between napping and global cognition or memory. Additionally, these findings were not modified by individual characteristics, including the mean age, the night sleep time (hours), and the percentage of women, no nappers, and people in the less nighttime sleep duration category.

Napping is a well-established sedentary behavior in many countries and a more common behavior as individuals become older. Therefore, this behavior has been traditionally studied to elucidate its positive and negative effects on health [9]. Although previous research has suggested both positive and negative associations of napping with cognitive function [37, 43, 46–50, 52–54], our data indicate no cross-sectional association between napping and specific cognitive functions, including global cognition and memory. Additionally, a U-shape association has been suggested to describe the cross-sectional relationship between napping and cognitive function [33, 53], which we cannot confirm because the duration and frequency of napping were not reported by all the included studies.

The longitudinal association between napping and cognitive functions has been considered a two-way relationship. While a negative effect has been reported for napping on cognitive functions, patients with cognitive decline have also been reported to tend to sleep more during the day as cognitive decline advances [50]. When analyzing the longitudinal studies, we did not find an association of napping with global cognition or memory in the general population. These results suggest that napping does not seem to modify cognitive functions in longitudinal studies regardless of whether the trajectory of cognition is not the same for all adults, which has been previously described to have a negative influence on the cognitive function and sleep disturbances associated with aging. [55, 56]

Although our data indicate no association of napping with cognitive functions, several aspects might be considered when analyzing this relationship. The high prevalence of chronic diseases among the elderly population may be related to daily sleepiness induced by medication or fatigue [57, 58]. Additionally, the frequency of napping might be considered since some differences might be found when napping several short times during the day instead of taking an isolated longer nap. In this sense, naps may have a compensatory function when night sleep is fragmented or not completely restorative [59]. Finally, the relationship between napping as a sedentary behavior and isolation might be considered since aging is associated with an increase in the depression incidence, which may be fostered by inactivity and isolation [60, 61]. Unexpectedly, this systematic review and meta-analysis could not address these issues. Further studies addressing these issues are needed to better understand the relationship between napping and cognitive functions.

Some neurobiological mechanisms have been proposed to explain the positive effects of napping on cognition. Awake periods increase amyloid-B accumulation in the brain extracellular space, which is a peptide that interferes with synaptic activity and may be cleared during sleep periods [62]. Additionally, reduced neural activity during napping is proposed to relax oxidative processes and vascular demands, fostering the clearance of waste products [49]. Last, sleep periods have been described as essential to consolidate memory. Conversely, negative effects of napping have also been reported. Napping has been related to higher levels of inflammatory markers, including IL-6 levels, which may induce cognitive impairment [63]. Furthermore, napping after lunch may disrupt circadian rhythms at the day-point of best coordination and fastest reaction time [50]. This systematic review and meta-analysis could not confirm any of these positive or negative statements.

This systematic review and meta-analysis suffers from specific limitations that may be highlighted. First, the considerable heterogeneity reported in meta-analyses might reduce the stability of our results and conclusions. Second, language restrictions could generate some risk of bias in the results. Third, the studies included in this systematic review and meta-analysis may differ in the methods used to collect data on napping and cognitive function; additionally, substantial inconsistency was found for the analyses. Fourth, cause-effect relationships could not be inferred from the cross-sectional analysis. Fifth, the influence of some important variables and nap characteristics could not be explored, as they were not reported by the original studies. Sixth, whether napping is an underlying sleep disorder or comorbidity or whether the estimated associations differ based on comorbidities could not be determined in this systematic review and meta-analysis. Seventh, nap determination was based mostly on questionnaires; therefore, differences from the actigraphy data could not be determined. Eight, only data for global cognition and memory could be explored, information on other cognitive domains would be of interest. Ninth, publication bias and modification in the pooled ORs were detected after sensitivity analyses, therefore our results might be considered with caution. Finally, only one study reported more than one follow-up measurement; thus, we could not examine the trajectory of the relationship between napping and cognitive decline over time.

This systematic review and meta-analysis following statistical procedures, was proposed to cover part of the need to clarify the relationships between napping and health outcomes. [18] Our data confirm the lack of an association between napping and global cognition and memory. Additionally, such associations are not modified by individual characteristics, including age and the percentage of women in the sample, or sleep characteristics. This information might be considered when providing lifestyle recommendations to adults with and without cognitive complaints. Further studies considering key variables that may influence such associations, including depression, chronic diseases, and the number of breaks for napping, are needed.

## Supplementary Information

**Additional file 1:** **Supplementary Table 1.** PRISMA checklist. **Supplementary Table 2.** Search strategy for Medline. **Supplementary** **Figure 1. **Preferred Reporting Items for Systematic Reviews flowchart. **Supplementary Table 3.** Characteristics of the Cross-Sectional Studies Included in the Systematic Review and Meta-Analysis on the Association Between Napping and Cognition Parameters. **Supplementary Table 4**. Characteristics of the Longitudinal Studies Included in the Systematic Review and Meta-Analysis on the Association Between Napping and Cognition Parameters. **Supplementary** **Table 5.** Covariates used to adjust the analyses reported by the included studies. **Supplementary** **Table 6.** Risk of bias of cross-sectional and longitudinal included studies. **Supplementary Table 7. **Inconsistence and heterogeneity estimations for DerSimoniand and Lair and Hartung-Knapp-Sidik-Jonkman random effects methods. **Supplementary Table 8.** Meta-regression of napping and cognition domains by percentage of females and mean age of included studies. **Supplementary Table 9.** Meta-regression of napping and cognition domains by percentage of no nappers, percentage of people included in the less nighttime sleep duration category and mean night sleep time (hours) included studies. **Supplementary Table 10.** Sensitivity analyses by removing studies one by one for cross-sectional analysis. **Supplementary Table 11.** Sensitivity analyses by removing studies one by one for longitudinal analysis. **Supplementary Table 12.** Meta-bias for the association between IMT and cognitive function domains.

## Data Availability

Data from this paper are available from other researches upon request. Alvarez-Bueno C should be contacted if someone wants to request the data from this study.
