# Supplementary material for: Napping and cognitive decline: a systematic review and meta-analysis of observational studies
Source: BMC Geriatr. 2022 Sep 15;22:756. doi: 10.1186/s12877-022-03436-2 (PMC9479293; doi:10.1186/s12877-022-03436-2)
Supplement: Supplementary file 1 — Additional file 1: Supplementary Table 1. PRISMA checklist. Supplementary Table 2. Search strategy for Medline. Supplementary Figure 1. Preferred Reporting Items for Systematic Reviews flowchart. Supplementary Table 3. Characteristics of the Cross-Sectional Studies Included in the Systematic Review and Meta-Analysis on the Association Between Napping and Cognition Parameters. Supplementary Table 4. Characteristics of the Longitudinal Studies Included in the Systematic Review and Meta-Analysis on the Association Between Napping and Cognition Parameters. Supplementary Table 5. Covariates used to adjust the analyses reported by the included studies. Supplementary Table 6. Risk of bias of cross-sectional and longitudinal included studies. Supplementary Table 7. Inconsistence and heterogeneity estimations for DerSimoniand and Lair and Hartung-Knapp-Sidik-Jonkman random effects methods. Supplementary Table 8. Meta-regression of napping and cognition domains by percentage of females and mean age of included studies. Supplementary Table 9. Meta-regression of napping and cognition domains by percentage of no nappers, percentage of people included in the less nighttime sleep duration category and mean night sleep time (hours) included studies. Supplementary Table 10. Sensitivity analyses by removing studies one by one for cross-sectional analysis. Supplementary Table 11. Sensitivity analyses by removing studies one by one for longitudinal analysis. Supplementary Table 12. Meta-bias for the association between IMT and cognitive function domains. [file 12877_2022_3436_MOESM1_ESM.docx]

| **Section and Topic** | **Item #** | **Checklist item** | **Location where item is reported** | |
| --- | --- | --- | --- | --- |
| **TITLE** | | |  |  |
| Title | 1 | Identify the report as a systematic review. | 1 page | |
| **ABSTRACT** | | |  |  |
| Abstract | 2 | See the PRISMA 2020 for Abstracts checklist. | 2 page | |
| **INTRODUCTION** | | |  |  |
| Rationale | 3 | Describe the rationale for the review in the context of existing knowledge. | 3-4 page | |
| Objectives | 4 | Provide an explicit statement of the objective(s) or question(s) the review addresses. | 4 page | |
| **METHODS** | | |  |  |
| Eligibility criteria | 5 | Specify the inclusion and exclusion criteria for the review and how studies were grouped for the syntheses. | 5 page | |
| Information sources | 6 | Specify all databases, registers, websites, organisations, reference lists and other sources searched or consulted to identify studies. Specify the date when each source was last searched or consulted. | 4-5 page | |
| Search strategy | 7 | Present the full search strategies for all databases, registers and websites, including any filters and limits used. | Supplement | |
| Selection process | 8 | Specify the methods used to decide whether a study met the inclusion criteria of the review, including how many reviewers screened each record and each report retrieved, whether they worked independently, and if applicable, details of automation tools used in the process. | 5 page | |
| Data collection process | 9 | Specify the methods used to collect data from reports, including how many reviewers collected data from each report, whether they worked independently, any processes for obtaining or confirming data from study investigators, and if applicable, details of automation tools used in the process. | 5-6 page | |
| Data items | 10a | List and define all outcomes for which data were sought. Specify whether all results that were compatible with each outcome domain in each study were sought (e.g. for all measures, time points, analyses), and if not, the methods used to decide which results to collect. | 5-6 page | |
|  | 10b | List and define all other variables for which data were sought (e.g. participant and intervention characteristics, funding sources). Describe any assumptions made about any missing or unclear information. | 5-6 page | |
| Study risk of bias assessment | 11 | Specify the methods used to assess risk of bias in the included studies, including details of the tool(s) used, how many reviewers assessed each study and whether they worked independently, and if applicable, details of automation tools used in the process. | 5-6 page | |
| Effect measures | 12 | Specify for each outcome the effect measure(s) (e.g. risk ratio, mean difference) used in the synthesis or presentation of results. | 6-7 page | |
| Synthesis methods | 13a | Describe the processes used to decide which studies were eligible for each synthesis (e.g. tabulating the study intervention characteristics and comparing against the planned groups for each synthesis (item #5)). | 6-7 page | |
|  | 13b | Describe any methods required to prepare the data for presentation or synthesis, such as handling of missing summary statistics, or data conversions. | 6-7 page | |
|  | 13c | Describe any methods used to tabulate or visually display results of individual studies and syntheses. | 6-7 page | |
|  | 13d | Describe any methods used to synthesize results and provide a rationale for the choice(s). If meta-analysis was performed, describe the model(s), method(s) to identify the presence and extent of statistical heterogeneity, and software package(s) used. | 6-7 page | |
|  | 13e | Describe any methods used to explore possible causes of heterogeneity among study results (e.g. subgroup analysis, meta-regression). | 6-7 page | |
|  | 13f | Describe any sensitivity analyses conducted to assess robustness of the synthesized results. | 6-7 page | |
| Reporting bias assessment | 14 | Describe any methods used to assess risk of bias due to missing results in a synthesis (arising from reporting biases). | 6-7 page | |
| Certainty assessment | 15 | Describe any methods used to assess certainty (or confidence) in the body of evidence for an outcome. | 6-7 page | |
| **RESULTS** | | |  |  |
| Study selection | 16a | Describe the results of the search and selection process, from the number of records identified in the search to the number of studies included in the review, ideally using a flow diagram. | 7 page | |
|  | 16b | Cite studies that might appear to meet the inclusion criteria, but which were excluded, and explain why they were excluded. | Flow chart | |
| Study characteristics | 17 | Cite each included study and present its characteristics. |  | |
| Risk of bias in studies | 18 | Present assessments of risk of bias for each included study. | Supplement | |
| Results of individual studies | 19 | For all outcomes, present, for each study: (a) summary statistics for each group (where appropriate) and (b) an effect estimate and its precision (e.g. confidence/credible interval), ideally using structured tables or plots. | Forest plot | |
| Results of syntheses | 20a | For each synthesis, briefly summarise the characteristics and risk of bias among contributing studies. | 8 page | |
|  | 20b | Present results of all statistical syntheses conducted. If meta-analysis was done, present for each the summary estimate and its precision (e.g. confidence/credible interval) and measures of statistical heterogeneity. If comparing groups, describe the direction of the effect. | 8-9 page | |
|  | 20c | Present results of all investigations of possible causes of heterogeneity among study results. | 8-9 page | |
|  | 20d | Present results of all sensitivity analyses conducted to assess the robustness of the synthesized results. | 8-9 page | |
| Reporting biases | 21 | Present assessments of risk of bias due to missing results (arising from reporting biases) for each synthesis assessed. | 8-9 page | |
| Certainty of evidence | 22 | Present assessments of certainty (or confidence) in the body of evidence for each outcome assessed. | 8-9 page | |
| **DISCUSSION** | | |  |  |
| Discussion | 23a | Provide a general interpretation of the results in the context of other evidence. | 9 page | |
|  | 23b | Discuss any limitations of the evidence included in the review. | 11 page | |
|  | 23c | Discuss any limitations of the review processes used. | 11 page | |
|  | 23d | Discuss implications of the results for practice, policy, and future research. | 11 page | |
| **OTHER INFORMATION** | | |  |  |
| Registration and protocol | 24a | Provide registration information for the review, including register name and registration number, or state that the review was not registered. | 4 page | |
|  | 24b | Indicate where the review protocol can be accessed, or state that a protocol was not prepared. | 4 page | |
|  | 24c | Describe and explain any amendments to information provided at registration or in the protocol. | Not needed | |
| Support | 25 | Describe sources of financial or non-financial support for the review, and the role of the funders or sponsors in the review. | 12 page | |
| Competing interests | 26 | Declare any competing interests of review authors. | 12 page | |
| Availability of data, code and other materials | 27 | Report which of the following are publicly available and where they can be found: template data collection forms; data extracted from included studies; data used for all analyses; analytic code; any other materials used in the review. | Not reported | |

*From:*  Page MJ, McKenzie JE, Bossuyt PM, Boutron I, Hoffmann TC, Mulrow CD, et al. The PRISMA 2020 statement: an updated guideline for reporting systematic reviews. BMJ 2021;372:n71. doi: 10.1136/bmj.n71

For more information, visit: <http://www.prisma-statement.org/>

**Supplementary Table 2:** Search strategy for Medline

| “napping”  OR  “siesta”  OR  “nap”  OR  “nap sleep”  OR  “nap time”  OR  “day sleep”  OR  “daytime sleep”  OR  “daytime nap”  OR  “daytime napping”  OR  “day time sleep”  OR  “day time nap”  OR  “day time napping”  OR  “day-time sleep”  OR  “day-time nap”  OR  “day-time napping” | AND | “elderly”  OR  “older adults”  OR  “older adult”  OR  “middle-aged adults”  OR  “aged individuals”  OR  “aged adults”  OR  senior*  OR  “ancient”  OR  “ageing” | AND | “cognition”  OR  “executive”  OR  “executive function”  OR  “cognitive control”  OR  “memory”  OR  “attention”  OR  “metacognition”  OR  “life skills”  OR  “goal setting”  OR  “problem solving”  OR  “self-regulation”  OR  “brain development”  OR  “brain health”  OR  “neural” |
| --- | --- | --- | --- | --- |

**Supplementary** **Figure 1:** Preferred Reporting Items for Systematic Reviews flowchart.

**Supplementary Table 3**. Characteristics of the Cross-Sectional Studies Included in the Systematic Review and Meta-Analysis on the Association Between Napping and Cognition Parameters

|  |  | **Subjects Characteristics** | | | **Sleep characteristics** | | | **Outcome** | |
| --- | --- | --- | --- | --- | --- | --- | --- | --- | --- |
| **References** | **Country** | **n, female (%)** | **Age** | **Depressive s (%)** | **Device** | **Night-time sleep duration (hours)** | **Nap duration (min) or frequency** | **Cognitive measurement** | **Cognitive domain** |
| Auyeung et al., 2013 | China | 2945 (40.85) | 73.89 (4.99) | GDS≥8=4.9% | Questionnaire | 7.96 (1.39) | No nappers: 44%  Nappers: 56% | MMSE | Global cognitive function |
| Blackwell et al., 2006 | USA | 2932 (100) | 83.5 (3.7) | GDS mean: 2.3 (2.5) | Actigraphy | 6.7 (1.3)  <5 h: 8.6% | 75.0 (64.2) | MMSE  TMT-B | Global cognitive function |
| Cai et la, 2021 | China | 2214 (58.80) | 70.40 (8.22)-71.09 (7.62) | NA | Questionnaire | 6.54 (1.49)-6.61 (1.51) | No nappers: 30.71%  Nappers: 69.29% | MMSE  MoCA  Neuropsychological Test Battery | Global cognitive function  Digit span, auditory verbal learning, associative learning, visual retention, language fluency, mapping and a test with blocks |
| Chiu et al., 2016 | Taiwan | 2413 (54.66) | 74.1 (6.5) | CESD-10 mean: 4.8 (4.2) | Questionnaire | <6.5 h: 54.7-62.5%  6.5-8.5 h: 31.4-36.2%  >8.5 h: 6.1-9.1% | 49.5 (47.1)-55.4 (48.3) | MMSE | Global cognitive function |
| Coelho et al., 2020 | Canada | 75 (80) | 74.6 (9) | NA | Questionnaire | NA | NA | Inhibit, shift, emotional control, and self-monitor  Initiate, working memory, plan/organize, task monitor, and organization of materials  Stroop and Snap Modified version of  Wisconsin card sort  Tower of Hanoi and Mega and Lego blocks | Behavioural Regulation index  Metacognitive Index  Inhibition  Executive functions |
| Cross et al., 2015 | Australia | 133 (51.1) | 65.5 (8.4) | GDS-30 mean: 9.23 (7.7)-10.76 (8.14) | Actigraphy  Questionnaire | 7.53 (1.13)- 7.61 (0.80) | 72.5 (37.4)  No nappers:16.54%  Nappers: 83.46% | MMSE  TMT-A  TMT-B  LM-II  COWAT  Stroop | Global cognitive function  Psychomotor speed  Set shifting  Verbal memory  Verbal fluency  Inhibition |
| Fang et al., 2018 | Taiwan | 1724 (52.15) | 73.22 (4.63) | CESD-10 mean: 4.39 (3.94) | Questionnaire | 5.98 (1.45)-6.38 (1.67) | No nappers: 30.4-41.81%  Short nappers: 33.02-42.83%  Long nappers: 25.18-27.67% | MMSE | Global cognitive function |
| Frisoni et al., 1996 | Italy | 223 (67.7) | 79.97 (4.20) | NA | Questionnaire | NA | No nappers: 76.2%  Nappers 1-2 d/w: 6.3%  Nappers 3-4 d/w: 3.1%  Nappers >4d/w: 14.4% | MMSE | Global cognitive function |
| Li et al., 2017 | China | 2974 (43.5) | 71.4 (5.4) | CESD-10 mean: 8.8 (6.3) | Questionnaire | 6.2 (2.1)  <5 h: 21.4%  5-6.9: 34.3%  7-8.9: 35.2%  >9 h:9.1% | 62.9 (41.8)  No nappers: 42.26%  Short nappers: 9.61%  Moderate nappers: 35.00%  Extended nappers: 13.11% | Telephone Interview of Cognitive Status  Figure drawing  Word recall | Global cognitive function  Memory |
| Lin et al., 2018 | China | 10740 (53.4) | 69.5 (7.6) | PHQ-9 mean: 1.5 (2.7) | Questionnaire | <5 h: 13.0%  5–6.9 h: 38.8%  7–8.9 h: 41.8%  ≥9 h: 6.4% | 0 min 46.2%  1–30 min 18.0%  31–60 min 18.8%  >60 min 17.0% | MMSE | Global cognitive function |
| Ohayon and Vecchierin., 2002 | France | 1026 (59.8) | >60 | NA | Questionnaire | 7 (1.28)-7.12 (2.02)  <5 h: 11%  5-7 h: 19.30%  7-8.30 h: 56.25%  >8.30 h: 13.45% | None: 73.8%  Intentional: 22%  Unintentional: 4.2% | Mac-Nair-R  MMSE | Cognitive difficulties  Global cognitive function |
| Owusu et al., 2019 | USA | 2549 (57.1) | >60 | PHQ-2 mean: 0.84 (0.03) | Questionnaire | 6.99 (0.04) | Short nappers: 45%  Moderate nappers: 34.9%  Long nappers: 20.1% | Clock drawing test  Immediate word recall  Delayed word recall | Global cognitive function  Memory  Memory |
| Qian et al., 2020 | China | 4579 (NA) | >60 | NA | Questionnaire | NA | No nappers: 94.2-98.4%  Nappers: 1.6-5.8% | Abbreviated Mental Test | Global cognitive function |
| Sagherian and Rose, 2020 | USA | 308 (48.38) | 74.07 (5.45) | PHQ-2: 5.56% | Questionnaire | 6.87 (1.27) | No nappers: 57.52%  Brief naps (≤ 45 min): 23.86%  Prolonged naps (> 45 min): 18.63% | Clock drawing test  Orientation  Delayed Word Recall | Global cognitive function  Orientation  Memory |
| Wang et al., 2022 | China | 9218 (51.1) | 61.5 (8.7) | CESD-10: 33.6% | Questionnaire | <5 h: 22%  5-6 h: 15%  6-7 h: 17.8%  7-8 h: 19.1%  >9 h: 26.1% | Non-nappers: NA  Short nappers: NA  Moderate nappers: NA  Extended nappers: NA | Mental Status Score  Word recall | Global cognitive function  Memory |
| Xie et al., 2022 | China | 2665 (32.72) | 71.8 (5.4) | CESD-10 mean: 6.8 (5.8) | Questionnaire | <5h: 15.05%  5-7h: 39.85%  7-9h: 38.42%  >9h: 3.33% | No nappers: 32.83%  <30 min: 8.85%  30-90 min: 45.25%  >90 min: 13.05% | MMSE | Global cognitive function |
| Xin et al., 2020 | China | 7469 (50.0) | 67.95 (NA) | NA | Questionnaire | <6 h: 34.3%  6-8 h: 35.3%  >8 h: 30.4% | 40.8 (0.5)  No nappers (0 min): 40.5%  Moderate nappers (1-60 min): 39.4%  Long nappers (>60 min): 20.1% | Rowe and Kahn’s | Cognitive functioning |
| Xu et al., 2011 | China | 28670 (72.47) | 61.5 (7.0)-65.3 (7.2) | NA | Questionnaire | 6.82 (1.48)-6.94 (1.32) | No nappers: 57.9-61%  Nappers: 39-42.1% | Word recall test | Memory |

NA: not available; MMSE: Mini-Mental State Examination; TMT: Trail Making Test; LM-II: Logical Memory II; COWAT: Controlled Oral Word Associated Test; SLMUS: St. Louis University Mental Status Examination

**Supplementary Table 4**. Characteristics of the Longitudinal Studies Included in the Systematic Review and Meta-Analysis on the Association Between Napping and Cognition Parameters

|  |  |  | **Subjects Characteristics** | | | **Sleep characteristics** | | | | **Outcome** | | |
| --- | --- | --- | --- | --- | --- | --- | --- | --- | --- | --- | --- | --- |
| **References** | **Country** | **Follow-up** | **n, female (%)** | **Age** | **Depressive s (%)** | **Device** | **Night-time sleep duration** | **Nap time (min)** | **Cognitive measurement** | | **Cognitive domain** |  |
| Cox et al., 2019 | UK | 2004-2007 to 2011-2013 | 1091 (46.29) | 69.52 (0.83) | HADS, mean: 7.67 (4.51) | Questionnaire | 6.09 (1.28) | 10 (37.5) | Matrix Reasoning and Block Design  Symbol Search and Digit-Symbol Substitution tests  Logical Memory, Verbal Paired Associates and Digit Span Backward test | | Visuospatial reasoning  Processing speed  Memory |  |
| Dzierzewski et la., 2014 | USA | 6 months | 192 (97.4) | 73.8 (9.4) | GDS-5 mean: 1.43 (1.37) | Actigraphy | NA | NA | MMSE | | Global cognitive function |  |
| Keage et al., 2012 | UK | 1991-1993 to 2001-2003 | 2012 (53) | 64-94 | NA | Questionnaire | <6.5 h: 20%  >6.5- <8.5 h: 62%  >8.5 h: 18% | No nappers: 38%  <60 min: 42%  >60 min: 20% | MMSE | | Global cognitive function |  |
| Kitamura et al., 2021 | Japan | 2011-2013 to 2016-2018 | 389 (59.12) | 74.6 (6.4) | NA | Questionnaire | <6 h: 14.5-20.9%  6-6.9 h: 23.9-28.3%  7-7.9 h: 31.7-32.7%  8-8.9 h: 13.9-22%  >9 h: 5.2-6.9% | No nappers:32.7-43.9%  1-29 min: 22-27.4%  30-59 min: 15.2-24.5%  >60 min: 13.5-20.8% | HDS-R | | Global cognitive function |  |
| Leng et al., 2019 | USA | 2003-2005 to 2014-2016 | 2751 (0) | <30 min: 75.3 (5.1)  30-59 min: 76.1 (5.1)  60-119 min: 76.6 (5.6)  >120min: 77.5 (5.7) | <30 min: 1.3 (1.7)  30-59 min: 1.8 (2.2)  60-119 min: 1.8 (2.1)  >120min: 2.3 (2.4) | Actigraphy | <6 h: 31.26%  6-8 h: 61.98%  >8 h: 6.76% | <30 min: 38.57%  30-59 min: 28.90%  60-119 min: 23.33%  >120 min: 9.20% | MMSE  TMT B | | Global cognitive function Sequencing, visual scanning, and executive function |  |
| Li et al., 2018 | China | 2011-2013 | 3037 (46.2) | 66.4 (5.4) | CES-10, mean: 8.1 (6.0) | Questionnaire | 6.1 (2.0) | 62.7 (60)  Non-nappers: 42.5%  Short nappers: 9.05%  Moderate nappers: 35.9%  Extended nappers: 12.54% | Figure drawing  Word recall | | Global cognitive function Memory |  |
| Sha et al., 2019 | China | 2011-2013 | 3584 (47.18) | 66.59 (5.50) | 8.56 (6.20) | Questionnaire | <5 h: 19.2%  5-7 h: 35.4%  7-9 h: 37.5%  >9 h: 14.5% | No nappers: 44.8%  <30 min: 9.3%  30-90 min: 31.3%  >90 min: 14.5% | TICS and Figure drawing test  Word recall | | Global cognitive function Memory |  |

NR: not reported; MMSE: Mini-Mental State Examination; TMT: Trail Making Test; MoCA; TICS: Telephone Interview of Cognitive Status; HDS-R: revised Hasegawa’s dementia scale

**Supplementary** **Table 5:** Covariates used to adjust the analyses reported by the included studies.

| Reference | Covariates included in the analysis |
| --- | --- |
| Auyeung et al., 2013 | Age, gender, MMSE score, years of education, smoking habit, regular alcohol, tea and coffee consumption, habitual snoring, depression (GDS≥8), use of psychotropic medications, diabetes mellitus, hypertension, stroke, chronic obstructive pulmonary disease, and coronary heart disease |
| Blackwell et al., 2006 | Age, race, depression, education, BMI, health status, history of stroke, history of hypertension, functional status, smoking, alcohol use, caffeine, antidepressant use, and physical activity |
| Cai et al., 2020 | Unadjusted |
| Chiu et al., 2016 | Age, sex, BMI, marital status, education levels, stroke, hypercholesterolemia, diabetes mellitus, CESD-10, exercise habits, alcohol intake, and tobacco consumption |
| Coelho et al., 2020 | Unadjusted |
| Cross et al., 2015 | Unadjusted |
| Cox et al., 2019 | Unadjusted |
| Dzierzewski et al., 2014 | Unadjusted |
| Fang et al., 2018 | Age, sex, years of education, BMI, depressive symptoms (CESD‐10), perceived health status, bodily pain, history of chronic disease, exercise and sleep characteristics (total sleep time, sleep efficiency, difficulty in initiating sleep, difficulty in maintaining sleep, early morning awakening, daytime sleepiness, hypnotic use and self‐reported sleep disorder). |
| Frisoni et al., 1996 | Unadjusted |
| Kitamura et al., 2021 | Age, baseline HDS-R score, sex, region (dummy variables), family environment, job status, histories of hypertension, cerebrovascular diseases, diabetes, alcohol consumption, smoking status, bedtime, duration of sleep, duration of nap (0, 1–29 min; 1, others), presence of sleep disturbance, and use of sleeping pills |
| Keage et al., 2012 | Sex, age at baseline, BMI classification (underweight <18.50, normal 18.50–24.99, overweight 25.00–29.99, and obese 30.00), education (defined as 9 years or less, and greater than 9 years) and cognition (not impaired, mildly impaired) at baseline |
| Leng et al., 2019 | Age, education, BMI, smoking, physical activity, depressive symptoms, history of stroke, coronary heart disease, hypertension, diabetes, sleep medication use and baseline 3MS score. |
| Li et al., 2018 | Age, gender, education, BMI, depression, IADL, morbidity and baseline cognition |
| Qian et al., 2020 | Age, gender. education level (primary education or below vs secondary schooling or above), marital status (living with a spouse vs not living with a spouse), monthly income (<1000 yuan, 1001–3000 yuan, or >3000 yuan), smoking (never, current, or former smoker), and alcohol consumption (yes vs no). |
| Sha et al., 2019 | Unadjusted |
| Xu et al., 2011 | Age, sex, employment, occupation, education, smoking, drinking, physical activity, tea consumption, self-rated health, waist circumference, HDL- and LDL-cholesterol, fasting plasma glucose and systolic blood pressure. |
| Xie et al., 2022 | Age, residence, education, marital status, night-time sleep duration, post-lunch napping duration, days with intensive physical activity, time with intensive/moderate/light physical activity, frequency of social activities, health status, physical disabilities, body pains, ADL, and CES-D-10 |

**Supplementary** **Table 6:** Risk of bias of cross-sectional and longitudinal included studies

|  | **1** | **2** | **3** | **4** | **5** | **6** | **7** | **8** | **9** | **10** | **11** | **12** | **13** | **14** | **Total** |
| --- | --- | --- | --- | --- | --- | --- | --- | --- | --- | --- | --- | --- | --- | --- | --- |
| **Cross-sectional studies** |  |  |  |  |  |  |  |  |  |  |  |  |  |  |  |
| Auyeung et al., 2013 | Y | Y | Y | Y | NR | - | - | Y | Y | - | Y | NR | - | Y | 8 |
| Blackwell et al., 2006 | Y | Y | N | Y | NR | - | - | Y | Y | - | Y | NR | - | Y | 7 |
| Cai et al., 2021 | Y | Y | Y | Y | NR | - | - | Y | Y | - | Y | NR | - | Y | 8 |
| Chiu et al., 2016 | Y | Y | Y | Y | NR | - | - | Y | Y | - | Y | NR | - | Y | 8 |
| Coelho et al., 2020 | Y | Y | Y | Y | NR | - | - | Y | Y | - | Y | NR | - | Y | 8 |
| Cross et al., 2015 | Y | Y | Y | Y | NR | - | - | Y | Y | - | Y | NR | - | Y | 8 |
| Fang et al., 2018 | Y | Y | Y | Y | NR | - | - | Y | Y | - | Y | NR | - | Y | 8 |
| Frisoni et al., 1996 | Y | Y | N | Y | NR | - | - | Y | Y | - | Y | NR | - | Y | 7 |
| Li et al., 2017 | Y | Y | Y | Y | NR | - | - | Y | Y | - | Y | NR | - | Y | 8 |
| Lin et al., 2018 | Y | Y | Y | Y | NR | - | - | Y | Y | - | Y | NR | - | Y | 8 |
| Ohayon and Vecchierin., 2002 | Y | Y | Y | Y | NR | - | - | Y | Y | - | Y | NR | - | Y | 8 |
| Owusu et al., 2019 | Y | Y | Y | Y | NR | - | - | Y | Y | - | Y | NR | - | Y | 8 |
| Qian et al., 2020 | Y | Y | Y | Y | NR | - | - | Y | Y | - | Y | NR | - | Y | 8 |
| Sagherian and Rose, 2020 | Y | Y | Y | Y | NR | - | - | Y | Y | - | Y | NR | - | Y | 8 |
| Wang et al., 2022 | Y | Y | Y | Y | NR | - | - | Y | Y | - | Y | NR | - | Y | 8 |
| Xie et al., 2022 | Y | Y | N | Y | NR | - | - | Y | Y | - | Y | NR | - | Y | 7 |
| Xin et al., 2020 | Y | Y | N | Y | NR | - | - | Y | Y | - | Y | NR | - | Y | 7 |
| Xu et al., 2011 | Y | Y | Y | Y | NR | - | - | Y | Y | - | Y | NR | - | Y | 8 |
| **Longitudinal studies** |  |  |  |  |  |  |  |  |  |  |  |  |  |  |  |
| Cox et al., 2019 | Y | Y | Y | Y | NR | NR | Y | Y | Y | N | Y | NR | N | Y | 9 |
| Dzierzewski et la., 2014 | Y | Y | Y | Y | NR | NR | Y | N | Y | Y | Y | NR | N | Y | 9 |
| Keage et al., 2012 | Y | Y | Y | Y | NR | NR | Y | Y | Y | Y | Y | NR | N | Y | 10 |
| Kitamura et al., 2021 | Y | Y | Y | Y | NR | NR | Y | Y | Y | Y | Y | NR | Y | Y | 11 |
| Leng et al., 2019 | Y | Y | Y | Y | NR | NR | Y | Y | Y | N | N | NR | Y | Y | 9 |
| Li et al., 2018 | Y | Y | Y | Y | NR | NR | Y | Y | Y | Y | Y | NR | Y | Y | 11 |
| Sha et al., 2019 | Y | Y | Y | Y | NR | NR | Y | Y | Y | N | Y | NR | Y | Y | 11 |

Y: yes; N: no; NR: not reported

**Supplementary Table 7:** Inconsistence and heterogeneity estimations for DerSimoniand and Lair and Hartung-Knapp-Sidik-Jonkman random effects methods.

|  | | | |  |  |
| --- | --- | --- | --- | --- | --- |
|  | **DerSimonian and Lair method** | | | **Hartung-Knapp-Sidik-Jonkman methods** | |
|  | | **Inconsistence (I2)** | **Heterogeneity (τ^2^)** | **Inconsistence (I2)** | **Heterogeneity (τ^2^)** |
| **Cross-sectional** | |  |  |  |  |
| Global cognition | | 71.8 | 0.0005 | 99.30 | 0.027 |
| Memory | | 90.2 | 0.0470 | 95.95 | 0.120 |
| **Longitudinal** | |  |  |  |  |
| Global cognition | | 67.4 | 0.0450 | 83.79 | 0.105 |
| Memory | | 43.4 | 0.0064 | 47.49 | 0.009 |

**Supplementary Table 8:** Meta-regression of napping and cognition domains by percentage of females and mean age of included studies.

|  | | | **% female** | | | | **Mean age** | | | |  |  |
| --- | --- | --- | --- | --- | --- | --- | --- | --- | --- | --- | --- | --- |
|  | | | **n** | **ß (95%CI)** | **p** | **n** | | **ß (95%CI)** | **p** | | |  |
|  |  | **Cross-sectional data** | | | | | | | |  |  |  |
| Global cognition | | | 7 | 0.003 (-0.009; 0.15) | 0.530 | 12 | | 0.006 (-0.022; 0.034) | 0.646 | | |  |
| Memory | | | 4 | -0.010 (-0.031; 0.117) | 0.188 | 6 | | 0.026 (-0.157; 0.209) | 0.715 | | |  |
|  |  | **Longitudinal data** | | | | | | | |  |  |  |
| Global cognition | | | 9 | -0.005 (-0.022; 0.012) | 0.511 | 9 | | 0.003 (-0.058; 0.065) | 0.919 | | |  |
| Memory | | | 4 | 0.004 (-0.034; 0.043) | 0.673 | 6 | | 0.010 (-0.168; 0.188) | 0.884 | | |  |
|  | | |  |  |  |  | |  |  | | |  |

**Supplementary Table 9:** Meta-regression of napping and cognition domains by percentage of no nappers, percentage of people included in the less nighttime sleep duration category and mean night sleep time (hours) included studies.

|  | | **No nappers (%)** | | | | **Less nighttime sleep (%)** | | | | | | | | **Mean night sleep (hours)** | | | | | | | | |  |
| --- | --- | --- | --- | --- | --- | --- | --- | --- | --- | --- | --- | --- | --- | --- | --- | --- | --- | --- | --- | --- | --- | --- | --- |
|  | | **n** | **ß (95%CI)** | **p** | | | **n** | | **ß (95%CI)** | | **p** | **n** | | | **ß (95%CI)** | | | **p** | | | | |  |
|  |  | **Cross-sectional data** | | | | | | | | | | | | | | | | | |  | | | |
| Global cognition | | 8 | -0.012 (-0.023; 0.000) | 0.042 | 6 | | | -0.005 (-0.014; 0.004) | | 0.225 | | | 5 | | | -0.082 (-0.232; 0.069) | 0.183 | | | |  |  |  |
| Memory | | 5 | -0.020 (-0.065; 0.025) | 0.247 | - | | | - | | - | | | 4 | | | -0.220 (-1.431; 0.991) | 0.516 | | | |  |  |  |
|  |  | **Longitudinal data** | | | | | | | | | | | | | | | | |  | | |  |  |
| Global cognition | | 5 | 0.014 (-0.025; 0.054) | 0.332 | | | 6 | | -0.001 (-0.047; 0.047) | | 0.991 | - | | | - | | | - | | | | |  |
| Memory | | - | - | - | | | 3 | | 0.003 (-0.100; 0.106) | | 0.785 | - | | | - | | | - | | | | |  |
|  | |  |  |  | | |  | |  | |  |  | | |  | | |  | | | | |  |

**Supplementary Table 10:** Sensitivity analyses by removing studies one by one for cross-sectional analysis.

| **Global cognition** | | | | |
| --- | --- | --- | --- | --- |
| **Reference** | **OR** | | **LL** | **UL** |
| Auyeung et al., 2013 | 1.017 | | 0.992 | 1.042 |
| Blackwell et al., 2006 | 1.017 | | 0.994 | 1.040 |
| Cai et al., 2020 | 1.015 | | 0.992 | 1.038 |
| Chiu et al., 2016 (men) | 1.094 | | 1.018 | 1.171 |
| Chiu et al., 2016 (women) | 1.094 | | 1.018 | 1.171 |
| Coelho et al., 2020 | 1.020 | | 0.996 | 1.043 |
| Cross et al., 2015 | 1.020 | | 0.996 | 1.044 |
| Fang et al., 2018 (short nappers) | 1.017 | | 0.994 | 1.040 |
| Fang et al., 2018 (long nappers) | 1.019 | | 0.993 | 1.040 |
| Frisoni et al., 1996 | 1.020 | | 0.995 | 1.043 |
| Qian et al., 2020 | 1.020 | | 0.997 | 1.044 |
| Sha et al, 2019 (men) | 1.013 | | 0.991 | 1.034 |
| Sha et al, 2019 (women) | 1.022 | | 0.999 | 1.046 |
| Xie et al., 2022 (nappers 30-90 min) | 1.014 | | 0.992 | 1.037 |
| Xie et al., 2022 (nappers <30 min) | 1.015 | | 0.992 | 1.038 |
| **Memory** | | | | |
| **Reference** | **OR** | **LL** | | **UL** |
| Cai et al., 2020 | 1.037 | 0.820 | | 1.254 |
| Cox et al., 2019 | 1.044 | 0.834 | | 1.255 |
| Cross et al., 2015 | 1.044 | 0.848 | | 1.240 |
| Sha et al, 2019 (men) | 1.013 | 0.808 | | 1.217 |
| Sha et al, 2019 (women) | 1.116 | 1.011 | | 1.221 |
| Xu et al., 2011 (daily nappers) | 1.066 | 0.822 | | 1.310 |
| Xu et al., 2011 (nappers 4-6 d/w) | 1.085 | 0.847 | | 1.324 |
| Xu et al., 2011 (nappers 1-3 d/w) | 1.064 | 0.834 | | 1.294 |

OR: odds ratio; LL: low limit; UL: upper limit

**Supplementary Table 11:** Sensitivity analyses by removing studies one by one for longitudinal analysis.

| **Global cognition** | | | |
| --- | --- | --- | --- |
| **Reference** | **OR** | **LL** | **UL** |
| Dzierzewski et al., 2014 | 0.932 | 0.728 | 1.136 |
| Keage et al., 2012 (short nappers) | 1.002 | 0.855 | 1.149 |
| Keage et al., 2012 (extended nappers) | 0.973 | 0.768 | 1.178 |
| Kitamura et al., 2021 (extended nappers) | 0.936 | 0.735 | 1.137 |
| Kitamura et al., 2021 (moderate nappers) | 0.949 | 0.745 | 1.153 |
| Kitamura et al., 2021 (short nappers) | 0.980 | 0.780 | 1.181 |
| Leng et al., 2019 (extended nappers) | 0.905 | 0.709 | 1.101 |
| Li et al., 2018 (extended nappers) | 0.918 | 0.715 | 1.123 |
| Li et al., 2018 (moderate nappers) | 0.936 | 0.718 | 1.154 |
| Li et al., 2018 (non nanppers) | 0.897 | 0.697 | 1.096 |
| Li et al., 2018 (short nappers) | 0.917 | 0.716 | 1.117 |
| Sha et al., 2019 (men) | 0.947 | 0.706 | 1.189 |
| Sha et al., 2019 (women) | 0.933 | 0.711 | 1.155 |
|  |  |  |  |
| **Memory** | | | |
| **Reference** | **OR** | **LL** | **UL** |
| Cox et al., 2019 | 1.071 | 0.951 | 1.191 |
| Li et al., 2018 (moderate nappers) | 1.023 | 0.940 | 1.119 |
| Li et al., 2018 (extended nappers) | 1.090 | 0.953 | 1.226 |
| Li et al., 2018 (short nappers) | 1.076 | 0.947 | 1.205 |
| Sha et al., 2019 (women) | 1.111 | 1.020 | 1.202 |
| Sha et al., 2019 (men) | 1.058 | 0.937 | 1.178 |

OR: odds ratio; LL: low limit; UL: upper limit

**Supplementary Table 12:** Meta-bias for the association between IMT and cognitive function domains.

|  | | |
| --- | --- | --- |
|  | **Coefficient** | **p** |
| **Cross-sectional** |  |  |
| Global cognition | 1.133 | 0.015 |
| Memory | 3.076 | 0.318 |
| **Longitudinal** |  |  |
| Global cognition | 0.294 | 0.817 |
| Memory | 3.672 | 0.128 |
